# Synthesis of New Macrocyclic Polyamides as Antimicrobial Agent Candidates

**DOI:** 10.3390/molecules171214510

**Published:** 2012-12-06

**Authors:** Osama I. Abd El-Salam, Mohamed A. Al-Omar, Ahmed A. Fayed, Eman M. Flefel, Abd El-Galil E. Amr

**Affiliations:** 1 Applied Organic Chemistry Department, National Research Center, Dokki 12622, Cairo, Egypt; 2 Pharmaceutical Chemistry Department, College of Pharmacy, King Saud University, Riyadh 11451, Saudi Arabia; 3 Pharmaceutical Chemistry Department, Drug Exploration & Development Chair (DEDC), College of Pharmacy, King Saud University, Riyadh 11451, Saudi Arabia; 4 Photochemistry Department, National Research Center, Dokki 12622, Cairo, Egypt

**Keywords:** macrocyclic polyamide candidates, amino acids, Schiff’s base, antimicrobial activity

## Abstract

A series of macrocyclic imides and Schiff-bases have been prepared via the cyclocondensation of pyridine-2,6-dicarbonyl dichloride (**1**) with L-ornithine methyl ester to give the corresponding macrocyclic bisester **2**. Treatment of **2** with hydrazine hydrate gave macrocyclic bisacid hydrazide **3**, which was used as starting material. Condensation of bishydrazide **3** with diacid anhydrides or aromatic aldehydes in refluxing acetic acid or ethanol gave the corresponding macrocyclic bisimides **4**, **5a**,**b** and macrocyclic bis- hydrazones **6a**–**j**, respectively. The structure assignments of the new compounds were based on chemical and spectroscopic evidence. The antimicrobial screening showed that many of these newly synthesized compounds have good antimicrobial activities, comparable to ampicillin and ketaconazole used as reference drugs.

## 1. Introduction

Among the different areas of macrocyclic chemistry, the synthesis and complexing properties of azacrown compounds have been a subject of intensive exploration [1,2,3,4,5,6,7]. The chemical modification of antibacterial agents in order to generate novel macrocyclic compounds with better therapeutic properties is necessary because of the emergence of multidrug resistant bacteria [8]. On the other hand, peptides rarely function well as drugs due to their low bioavailability and rapid degradation within cells [9]. In our previous work, we reported the synthesis of some macrocyclic candidates from the reactions of dipicolinic acid with amino acids and their biological activity screening [10,11,12,13,14]. In particular, 2,6-peptidopyridines exhibited a general ionophoric potency [15] and were used for inventing novel thiocyanate-selective membrane sensors [16]. Recently, some new heterocyclic steroidal and macrocyclic derivatives have been studied as 5α-reductase inhibitors, antiviral and anti-tumor agents [17], aromatase and quinone reductase-2 inhibitors [18], anti-inflammatory [19], anticonvulsant [20] and antimicrobial agents [21,22]. In view of these observations and in continuation of our previous work in macrocyclic chemistry, we have now synthesized some new macrocyclic derivatives containing pyridine and amino acid moieties. Some of the synthesized compounds were screened for their antimicrobial activity compared to the reference drugs ampicillin and ketaconazole.

## 2. Results and Discussion

### 2.1. Chemistry

**Scheme 1:**
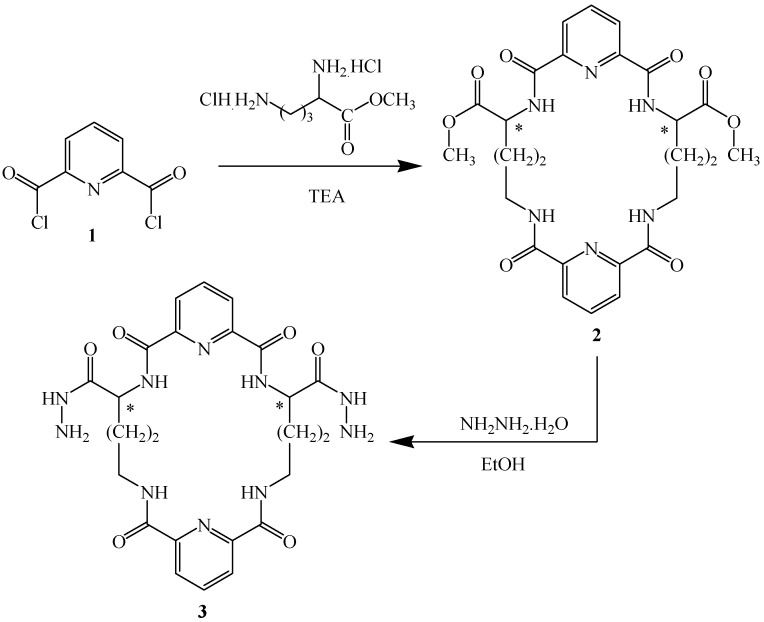
Synthetic pathway for starting compound **3**.

In our previous work, a series of chiral macrocyclic compounds were synthesized using macrocyclic bishydrazide derivative **3** [13], which was obtained from the corresponding ester **2** according to the published procedure [23,24] (Scheme 1).

**Scheme 2:**
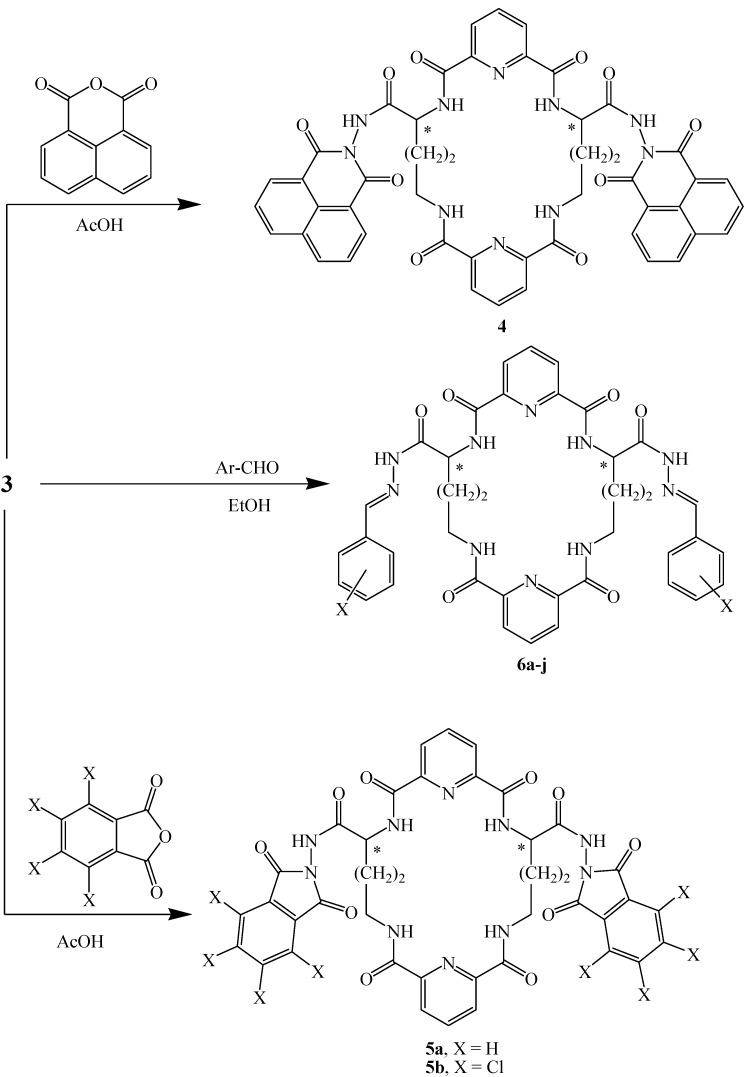
Synthetic pathway for compounds **4**, **5a**,**b** and **6a**–**j**.

Condensation in refluxing acetic acid of hydrazide **3** with selected acid anhydrides, namely 1,8-naphthalic anhydride, phthalic anhydride or 2,3,4,5-tetrachlorophthalic anhydride, afforded the corresponding tricyclobisdiimide derivatives **4** and **5a**,**b**, respectively (Table 1). Additionally, in light of the aforementioned biological interest in hydrazone derivatives [25,26,27], compound **3** was condensed with aromatic aldehydes in refluxing ethanol to afford the corresponding 4,20-di[oxo-(substituted phenyl)-carbohydrazonylmethyl)-3,8,16,21,27,28-hexaaza-2,9,15,22-tetraoxotricyclo-[3,21,1,1^10,14^]octacosa-1(26),10,12,14,23,25-hexene macrocyclic bishydrazones **6a**–**j** (Scheme 2 and Table 1). The structures of newly synthesized compounds **4**, **5a**,**b** and **6a**–**j** were confirmed by their IR, ^1^H-NMR, ^13^C-NMR and mass spectra.

**Table 1:** Melting points, crystallization solvents, yields, molecular formulae and molecular weights of compounds **4**, **5a**,**b** and **6a**–**j**.

Comp. No.	X	M.p. (°C)	Cryst. Solv.	Yield (%)	Molecular Formula (Mol. Wt.)
**4**	-	276–278	DMF/H_2_O	65	C_48_H_38_N_10_O_10_ (914.87)
**5a**	H	243–245	DMF/H_2_O	72	C_40_H_34_N_10_O_10_ (814.75)
**5b**	Cl	296–298	DMF/H_2_O	88	C_40_H_26_C_l8_N_10_O_10_ (1090.31)
**6a**	H	178–180	EtOH/Ether	85	C_38_H_38_N_10_O_6_ (730.77)
**6b**	3-Br	232–234	MeOH	79	C_38_H_36_Br_2_N_10_O_6_ (888.56)
**6c**	4-Br	254–256	Dioxane	87	C_38_H_36_Br_2_N_10_O_6_ (888.56)
**6d**	2,6-Cl_2_	198–200	EtO	68	C_38_H_34_Cl_4_N_10_O_6_ (868.55)
**6e**	3,4-Cl_2_	188–190	EtOH/Ether	78	C_38_H_34_Cl_4_N_10_O_6_ (868.55)
**6f**	2-Cl-6-F	168–170	AcOH/H_2_O	84	C_38_H_34_Cl_2_F_2_N_10_O_6_ (835.64)
**6g**	4-CH_3_	155–157	EtOH/H_2_O	82	C_40_H_42_N_10_O_6_ (758.82)
**6h**	2-OCH_3_	210–212	AcOH/H_2_O	90	C_40_H_42_N_10_O_8_ (790.82)
**6i**	4-OCH_3_	216–218	EtOH/H_2_O	80	C_40_H_42_N_10_O_8_ (790.82)
**6j**	3,4,5-(OCH_3_)_3_	235–257	AcOH/H_2_O	75	C_44_H_50_N_10_O_12_ (910.92)

### 2.2. Antimicrobial Testing

The newly synthesized compounds have been tested for their preliminary antimicrobial activity against the following microorganisms: Gram-positive bacteria, represented by *Bacillus subtilis* (NRRL B-543) and *Staphylococcus aureus* (NRRL B-313), Gram-negative bacteria, represented by *Escherichia coli* (NRRL B-558) and fungi, represented by *Candida albicans* (NRRL Y-477) and *Aspergillus niger* (NRRL Y-3). The results are summarized in Table 2.

**Table 2:** Antimicrobial activities of new synthesized compounds **4**, **5a**,**b** and **6a**–**j**.

Comp. No.	Inhibition zone (mm)				
	Gram +ve bacteria		Gram −ve bacteria	Fungi	
	*B. subtilis*	*Staph. aureus*	*E. coli*	*C. albicans*	*A. niger*
**3**	1.75	1.55	0.80	-	1.65
**4**	1.55	1.60	0.80	0.65	1.60
**5a**	1.35	1.75	0.60	0.75	1.90
**5b**	1.80	1.48	-	-	1.65
**6a**	1.20	1.70	-	-	1.85
**6b**	1.45	1.45	0.80	-	1.60
**6c**	0.90	1.30	-	0.65	1.70
**6d**	1.80	1.25	0.60	-	1.80
**6e**	1.75	0.85	-	-	1.75
**6f**	1.30	1.50	-	0.70	2.05
**6g**	0.85	1.30	0.75	0.65	1.95
**6h**	1.80	1.25	0.70	0.60	1.65
**6i**	1.70	1.20	0.75	-	1.55
**6j**	1.60	1.45	0.70	0.55	1.75
**Ampicillin**	1.15	1.30	0.75	-	-
**Ketaconazole **	-	-	-	0.80	2.30

From the data it appears that some of the synthesized compounds have modest antimicrobial activity. Except for **6c**, **6g** (against *B. subtilis*) and **6d**, **6e**, **6h**, **6i** (against *S. aureus*) the activities of tested compounds against these Gram-positive bacteria are slightly higher than that of the reference drug ampicillin. The activities of compounds **3**, **4**, **5a**,**b**, **6b**, **6d**, and **6g**–**j** against the Gram negative bacterium *E. coli* were all similar to the activity of the reference drug, while compounds **5b**, **6a**, **6c**, **6e **and **6f** are inactive against the same organism. In addition, the compounds tested against fungal organisms (*C. albicans* and *A.niger*) had lower activities than the reference drug nyastatin or, as in the case of compounds **5b**, **6a**, **6b**, **6d**, **6e** and **6i**, were inactive against *C. albicans* strain.

## 3. Experimental

### 3.1. General

Melting points were determined in open glass capillaries using in Electrothermal IA 9000 Series digital melting point apparatus (Electrothermal, Essex, UK) and are uncorrected. Elemental analyses were performed with all final compounds with an Elementar, Vario EL, Microanalytical Unit, National Research Centre, Cairo, Egypt and were in good agreement (*±*0.2%) with the calculated values. The IR spectra (KBr) were recorded on an FT IR-8201 PC spectrophotometer (Shimadzu, Tokyo, Japan). The ^1^H- and ^13^C-NMR spectra were measured with a Jeol 270 MHz spectrometer (FTGNM-EX 270, Tokyo, Japan) in DMSO-*d*_6_ as solvent. The chemical shifts were recorded relative to TMS. The Mass spectra (EI) were run at 70 eV with a Finnegan SSQ 7000 spectrometer (Thermo Instrument System Inc., Madison, WI, USA), *m/z* values are indicated in Dalton. TLC (silica gel, aluminum sheets 60F_254_, Merck, Darmstadt, Germany) was used for tracing the reactions. The starting material **3** was prepared according to the reported procedure [13] (Scheme 1). Antimicrobial screening was carried out in Department of Microbial Chemistry, National Research Center, Cairo, Egypt.

### 3.2. Chemistry

*Synthesis of Bisimidotricyclo-[3,23,1,1^11,15^]triaconta-1(28),11,13,15,25,27-hexene Derivatives* **4*** and* **5a**,**b**

A suspension of the hydrazide derivative **3** (0.554 g, 1 mmol) and 1,8-naphthalindicarboxylic anhydride, phthalic anhydride or 2,3,4,5-tetrachlorophthalic anhydride (2 mmol) in acetic acid (50 mL) was refluxed for 7 h. The solid was collected by filtration, washed with acetic acid and crystallized from dimethylformamide/water to give the corresponding macrocyclic octaamidodipyridyl derivatives **4** and **5a**,**b**, respectively.

*4,20-Di[oxo-2-(amino-3a,6-dihydro-1H-benzo[de]isoquinoline-1,3(2H)-dione)methyl]-3,8,16,21,27, 28-hexaaza-2,9,15,22-tetraoxotricyclo[3,21,1,1^10,14^]octacosa-1(26),10,12,14,22,25-hexene* (**4**): IR (KBr, cm^−1^): n 3340 (NH, amide), 1640 (C=N), 1660, 1534, 1320 (amide I, II and III). ^1^H-NMR (DMSO-*d*_6_): δ 1*.*32–1.36 (m, 4H, 2 *×* C*H*_2_), 1.54–1.62 (m, 4H, 2*×* C*H*_2_), 3.18–3.26 (m, 4H, 2*×* C*H*_2_), 4.38–4.54 (m, 2H, 2*×* C*H*-N), 7.75–8.10 (m, 12H, Ar-H), 8.24–8.36 (m, 6H, 2*×* pyr-*H*), 8.92 (m, 2H, 2*×* N*H*, exchangeable with D_2_O), 9.16 (m, 2H, 2*×* N*H*, exchangeable with D_2_O) and 10.08 (m, 2H, 2*×* N*H*, exchangeable with D_2_O). ^13^C-NMR: 27*.*62, 30.54, 38.54 (*6CH*_2_), 52.26 (2*CH*NH), 125.10, 125.16, 137.10, 137.14, 148.10, 148.24 (10pyr-*C*), 122.45, 124.98, 127.24, 128.95, 137.43, 137.76 (20Ar-C), 163.46, 169.50 (4*CO*NH), 157.88 (4*CO*-imide), 170.38 (*2CO*-amide). MS, *m/z* (%): 914 [M^+^, 24], 703 (14), 675 (45), 464 (72), 436 (35), 303 (76), 239 (100); Elemental analysis: calcd. for C_48_H_38_N_10_O_10_ (914.87): C, 63.02; H, 4.19; N, 15.31. found: C, 62.85; H, 4.14; N, 15.25.

*4,20-Di[oxo-2-(amino-1H-isoindole-1,3(2H)-dione)methyl]-3,8,16,21,27,28-hexaaza-2,9,15,22-tetra-oxotricyclo[3,21,1,1^10,14^]octacosa-1(26),10,12,14,22,25-hexene* (**5a**): IR (KBr, cm^−1^): n 3338 (NH, amide), 1642 (C=N), 1665, 1540, 1322 (amide I, II and III). ^1^H-NMR (DMSO-*d*_6_): δ 1*.*23–1.32 (m, 4H, 2*×* C*H*_2_), 1.48–1.58 (m, 4H, 2*×* C*H*_2_), 3.15–3.20 (m, 4H, 2*×* C*H*_2_), 4.42–4.52 (m, 2H, 2*×* C*H*-N), 7.65–7.80 (m, 8H, Ar-H), 8.30–8.38 (m, 6H, 2*×* pyr-*H*), 8.88 (m, 2H, 2*×* N*H*, exchangeable with D_2_O), 9.12 (m, 2H, 2*×* N*H*, exchangeable with D_2_O) and 10.15 (m, 2H, 2*×* N*H*, exchangeable with D_2_O). ^13^C-NMR: 27*.*65, 30.52, 38.58 (*6CH*_2_), 52.12 (2*CH*NH), 125.08, 125.12, 137.10, 137.18, 148.16, 148.32 (10pyr-*C*), 123.18, 131.78, 132.45 (12Ar-C), 163.62, 169.58 (4*CO*NH), 164.35 (4*CO*-imide), 170.15 (*2CO*-amide). MS, *m/z* (%): 815 [M^+^, 33], 653 (22), 522 (62), 492 (42), 464 (55), 436 (24), 189 (100); Elemental analysis: calcd. for C_40_H_34_N_10_O_10_ (814.75): C, 58.97; H, 4.21; N, 17.19. found: C, 58.92; H, 4.05; N, 17.15.

*4,20-Di[oxo-2-(amino-4,5,6,7-tetrachloro-1H-isoindole-1,3(2H)-dione)methyl]-3,8,16,21,27,28-hexa-aza-2,9,15,22-tetraoxotricyclo[3,21,1,1^10,14^]octacosa-1(26),10,12,14,22,25-hexene* (**5b**): IR (KBr, cm^−1^): n 3346 (NH, amide), 1645 (C=N), 1662, 1538, 1318 (amide I, II and III). ^1^H-NMR (DMSO-*d*_6_): δ 1*.*26–1.33 (m, 4H, 2*×* C*H*_2_), 1.38–1.55 (m, 4H, 2*×* C*H*_2_), 3.18–3.24 (m, 4H, 2*×* C*H*_2_), 4.46–4.54 (m, 2H, 2*×* C*H*-N), 8.26–8.34 (m, 6H, 2*×* pyr-*H*), 8.92 (m, 2H, 2*×* N*H*, exchangeable with D_2_O), 9.18 (m, 2H, 2*×* N*H*, exchangeable with D_2_O) and 10.08 (m, 2H, 2*×* N*H*, exchangeable with D_2_O). ^13^C-NMR: 27*.*55, 30.58, 38.60 (*6CH*_2_), 52.22 (2*CH*NH), 124.98, 125.05, 137.12, 137.16, 148.22, 148.30 (10pyr-*C*), 127.12, 132.96, 134.75 (12Ar-C), 163.58, 169.54 (4*CO*NH), 164.48 (4*CO*-imide), 170.26 (*2CO*-amide). MS, *m/z* (%):1090 [M^+^, 8], 789 (15), 492 (64), 464 (32), 436 (24), 324 (100); Elemental analysis: calcd. for C_40_H_26_Cl_8_N_10_O_10_ (1090.31): C, 44.06; H, 2.40; Cl, 26.01; N, 12.85. found: C, 44.00; H, 2.34; Cl, 25.94; N, 12.80.

*Synthesis of* *4,20-Di[oxo-(substituted phenyl)-carbohydrazonylmethyl)-3,8,16,21,27,28-hexaaza-2,9, 15,22-tetraoxotricyclo-[3,21,1,1^10,14^]octacosa-1(26),10,12,14,23,25-hexenes* **6a**–**j**

A mixture of the hydrazide derivative **3** (0.554 g, 1 mmol) and the appropriate aldehyde (2 mmol) in absolute ethanol (50 mL) was heated under reflux for 6 h. The solvent was evaporated under reduced pressure and the residue was solidified with ether. The solid was collected by filtration, washed with ether and crystallized from a proper solvent to afford the corresponding tricyclohexaazaoctacosabis- hydrazone derivatives **6a***–***j**, respectively. 

*4,20-Di[oxphenylcarbohydrazonylmethyl)-3,8,16,21,27,28-hexaaza-2,9,15,22-tetraoxotricyclo-[3,21, 1,1^10,14^]octacosa-1(26),10,12,14,23,25-hexene* (**6a**): IR (KBr, cm^−1^): n 3342 (NH, amide), 1646 (C=N), 1668, 1542, 1318 (amide I, II and III). ^1^H-NMR (DMSO-*d*_6_): δ 1*.*28–1.34 (m, 4H, 2*×* C*H*_2_), 1.50–1.60 (m, 4H, 2*×* C*H*_2_), 3.10–3.18 (m, 4H, 2*×* C*H*_2_), 4.44–4.56 (m, 2H, 2*×* C*H*-N), 7.45–7.68 (m, 12H, 2Ph-*H* + 2CH=N), 8.25–8.38 (m, 6H, 2*×* pyr-*H*), 8.86 (m, 2H, 2*×* N*H*, exchangeable with D_2_O), 8.94 (m, 2H, 2*×* N*H*, exchangeable with D_2_O) and 10.08 (m, 2H, 2*×* N*H*, exchangeable with D_2_O). ^13^C-NMR: 27*.*82, 30.40, 38.52 (*6CH*_2_), 52.05 (2*CH*NH), 147.12 (*2CH*=N), 125.16, 125.24, 137.15, 137.22, 148.20, 148.42 (10pyr-*C*), 123.85, 127.94, 129.38, 132.46 (12Ar-C), 163.66, 169.64 (4*CO*NH), 171.98 (*2CO*-hydrazone). MS, *m/z* (%):730 [M^+^, 6], 611 (34), 492 (45), 436 (55), 218 (100); Elemental analysis: calcd. for C_38_H_38_N_10_O_6_ (730.77): C, 62.46; H, 5.24; N, 19.17. found: C, 62.40; H, 5.17; N, 19.13.

*4,20-Di[oxo-3-bromophenylcarbohydrazonylmethyl)-3,8,16,21,27,28-hexaaza-2,9,15,22-tetraoxotri-cyclo[3,21,1,1^10,14^]octacosa-1(26),10,12,14,23,25-hexene* (**6b**): IR (KBr, cm^−1^): n 3343 (NH, amide), 1640 (C=N), 1660, 1542, 1318 (amide I, II and III). ^1^H-NMR (DMSO-*d*_6_): δ 1*.*26–1.34 (m, 4H, 2*×* C*H*_2_), 1.50–1.62 (m, 4H, 2*×* C*H*_2_), 3.18–3.24 (m, 4H, 2*×* C*H*_2_), 4.50–4.56 (m, 2H, 2*×* C*H*-N), 7.35–7.66 (m, 6H, Ar-*H*), 7.78 (s, 2H, Ar-H), 7.92 (s, 2H, 2CH=N), 8.24–8.35 (m, 6H, 2*×* pyr-*H*), 8.96 (m, 2H, 2*×* N*H*, exchangeable with D_2_O), 9.15 (m, 2H, 2*×* N*H*, exchangeable with D_2_O) and 10.16 (m, 2H, 2*×* N*H*, exchangeable with D_2_O). ^13^C-NMR: 27*.*70, 30.38, 38.52 (*6CH*_2_), 51.98 (2*CH*NH), 147.08 (*2CH*=N), 125.06, 125.12, 137.10, 137.16, 148.08, 148.16 (10pyr-*C*), 123.82, 127.94, 129.15, 132.18, 133.82, 135.32 (12Ar-C), 163.68, 169.76 (4*CO*NH), 172.14 (*2CO*-hydrazone). MS, *m/z* (%): 888 [M^+^, 23], 691 (45), 689 (76), 492 (100), 436 (55), 218 (82); Elemental analysis: calcd. for C_38_H_36_Br_2_N_10_O_6_ (888.56): C, 51.36; H, 4.08; N, 15.76. found: C, 51.30; H, 4.00; N, 15.71.

*4,20-Di[oxo-4-bromophenylcarbohydrazonylmethyl)-3,8,16,21,27,28-hexaaza-2,9,15,22-tetraoxotri-cyclo[3,21,1,1^10,14^]octacosa-1(26),10,12,14,23,25-hexene* (**6c**): IR (KBr, cm^−1^): n 3338 (NH, amide), 1644 (C=N), 1663, 1545, 1322 (amide I, II and III). ^1^H-NMR (DMSO-d_6_): δ 1*.*25–1.35 (m, 4H, 2*×* C*H*_2_), 1.52–1.64 (m, 4H, 2*×* C*H*_2_), 3.15–3.20 (m, 4H, 2*×* C*H*_2_), 4.54–4.58 (m, 2H, 2*×* C*H*-N), 7.55–7.78 (m, 10H, 2Ph-*H* + 2CH=N), 8.18–8.35 (m, 6H, 2*×* pyr-*H*), 8.88 (m, 2H, 2*×* N*H*, exchangeable with D_2_O), 8.96 (m, 2H, 2*×* N*H*, exchangeable with D_2_O) and 10.12 (m, 2H, 2*×* N*H*, exchangeable with D_2_O). ^13^C-NMR: 27*.*75, 30.42, 38.55 (*6CH*_2_), 52.15 (2*CH*NH), 146.98 (*2CH*=N), 125.18, 125.22, 137.05, 137.12, 148.18, 148.25 (10pyr-*C*), 123.80, 127.96, 129.34, 133.48 (12Ar-C), 163.65, 169.72 (4*CO*NH), 172.08 (*2CO*-hydrazone). MS, *m/z* (%): 888 [M^+^, 12], 691 (34), 689 (32), 492 (100), 436 (35), 218 (94); Elemental analysis: calcd. for C_38_H_36_Br_2_N_10_O_6_ (888.56): C, 51.36; H, 4.08; N, 15.76. found: C, 51.31; H, 4.01; N, 15.72.

*4,20-Di[oxo-2,6-dichlorophenylcarbohydrazonylmethyl)-3,8,16,21,27,28-hexaaza-2,9,15,22-tetraoxo-tricyclo[3,21,1,1^10,14^]octacosa-1(26),10,12,14,23,25-hexene* (**6d**): IR (KBr, cm^−1^): n 3344 (NH, amide), 1648 (C=N), 1660, 1541, 1319 (amide I, II and III). ^1^H-NMR (DMSO-d_6_): δ 1*.*26–1.32 (m, 4H, 2*×* C*H*_2_), 1.50–1.62 (m, 4H, 2*×* C*H*_2_), 3.18–3.22 (m, 4H, 2*×* C*H*_2_), 4.52–4.60 (m, 2H, 2*×* C*H*-N), 7.40–7.48 (m, 8H, 2Ph-*H* + 2CH=N), 8.18–8.32 (m, 6H, 2*×* pyr-*H*), 8.78 (m, 2H, 2*×* N*H*, exchangeable with D_2_O), 8.98 (m, 2H, 2*×* N*H*, exchangeable with D_2_O) and 10.15 (m, 2H, 2*×* N*H*, exchangeable with D_2_O). ^13^C-NMR: 27*.*66, 30.39, 38.65 (*6CH*_2_), 52.09 (2*CH*NH), 147.068 (*2CH*=N), 125.23, 125.25, 137.08, 137.10, 148.16, 148.22 (10pyr-*C*), 126.56, 127.48, 129.36, 133.52 (12Ar-C), 163.58, 169.68 (4*CO*NH), 172.15 (*2CO*-hydrazone). MS, *m/z* (%): 868 [M^+^, 8], 679 (18), 492 (58), 436 (42), 245 (100), 205 (78); Elemental analysis: calcd. for C_38_H_34_Cl_4_N_10_O_6_ (868.55): C, 52.55; H, 3.95; Cl, 16.33; N, 16.13. found: C, 52.50; H, 3.91; Cl, 16.28; N, 16.08.

*4,20-Di[oxo-3,4-dichlorophenylcarbohydrazonylmethyl)-3,8,16,21,27,28-hexaaza-2,9,15,22-tetraoxo-tricyclo[3,21,1,1^10,14^]octacosa-1(26),10,12,14,23,25-hexene* (**6e**): IR (KBr, cm^−1^): n 3346 (NH, amide), 1626 (C=N), 1662, 1539, 1322 (amide I, II and III). ^1^H-NMR (DMSO-*d*_6_): δ 1*.*28–1.35 (m, 4H, 2*×* C*H*_2_), 1.49–1.60 (m, 4H, 2*×* C*H*_2_), 3.24–3.26 (m, 4H, 2*×* C*H*_2_), 4.46–4.58 (m, 2H, 2*×* C*H*-N), 7.55–7.65 (m, 6H, 4H-Ar + 2CH=N), 7.86 (s, 2H, Ar-H), 8.18–8.26 (m, 6H, 2*×* pyr-*H*), 8.84 (m, 2H, 2*×* N*H*, exchangeable with D_2_O), 9.05 (m, 2H, 2*×* N*H*, exchangeable with D_2_O) and 10.18 (m, 2H, 2*×* N*H*, exchangeable with D_2_O). ^13^C-NMR: 27*.*34, 30.42, 37.98 (*6CH*_2_), 51.96 (2*CH*NH), 147.08 (*2CH*=N), 124.95, 125.05, 137.10, 137.14, 148.08, 148.12 (10pyr-*C*), 126.94, 129.45, 129.55, 132.65, 132.76, 134.68 (12Ar-C), 163.45, 169.72 (4*CO*NH), 171.88 (*2CO*-hydrazone). MS, *m/z* (%): 868 [M^+^, 12], 868 {M^+^+2, 5], 679 (22), 492 (25), 436 (56), 245 (78), 214 (100); Elemental analysis: calcd. for C_38_H_34_Cl_4_N_10_O_6_ (868.55): C, 52.55; H, 3.95; Cl, 16.33; N, 16.13. found: C, 52.48; H, 3.90; Cl, 16.29; N, 16.10.

*4,20-Di[oxo-2-chloro-6-fluorophenylcarbohydrazonylmethyl)-3,8,16,21,27,28-hexaaza-2,9,15,22-tetraoxo-tricyclo[3,21,1,1^10,14^]octacosa-1(26),10,12,14,23,25-hexene* (**6f**): IR (KBr, cm^−1^): n 3352 (NH, amide), 1618 (C=N), 1660, 1538, 1324 (amide I, II and III). ^1^H-NMR (DMSO-*d*_6_): δ 1*.*34–1.38 (m, 4H, 2*×* C*H*_2_), 1.44–1.58 (m, 4H, 2*×* C*H*_2_), 3.30–3.36 (m, 4H, 2*×* C*H*_2_), 4.50–4.57 (m, 2H, 2*×* C*H*-N), 7.45–7.85 (m, 8H, Ar-H + 2CH=N), 8.15–8.30 (m, 6H, 2*×* pyr-*H*), 8.86 (m, 2H, 2*×* N*H*, exchangeable with D_2_O), 9.10 (m, 2H, 2*×* N*H*, exchangeable with D_2_O) and 10.16 (m, 2H, 2*×* N*H*, exchangeable with D_2_O). ^13^C-NMR: 27*.*54, 30.36, 37.84 (*6CH*_2_), 52.04 (2*CH*NH), 147.12 (*2CH*=N), 125.12, 125.16, 137.16, 137.24, 147.96, 148.05 (10pyr-*C*), 113.68, 117.88, 124.82, 133.52, 134.56, 161.02 (12Ar-C), 163.62, 169.76 (4*CO*NH), 171.94 (*2CO*-hydrazone). MS, *m/z* (%): 834 [M^+^, 17], 8636 [M^+^+2, 6], 663 (18), 492 (15), 464 (8), 245 (62), 199 (100); Elemental analysis: calcd. for C_38_H_34_Cl_2_F_2_N_10_O_6_ (835.64): C, 54.62; H, 4.10; Cl, 8.49; 16.76. found: C, 54.58; H, 4.05; Cl, 8.43; 16.72.

*4,20-Di[oxo-4-methylphenylcarbohydrazonylmethyl)-3,8,16,21,27,28-hexaaza-2,9,15,22-tetraoxotri-cyclo[3,21,1,1^10,14^]octacosa-1(26),10,12,14,23,25-hexene* (**6g**): IR (KBr, cm^−1^): n 3340 (NH, amide), 1638 (C=N), 1660, 1552, 1324 (amide I, II and III). ^1^H-NMR (DMSO-*d*_6_): δ 1*.*32–1.38 (m, 4H, 2*×* C*H*_2_), 1.55–1.65 (m, 4H, 2*×* C*H*_2_), 2.25 (s, 6H, 2 × CH_3_), 3.18–3.24 (m, 4H, 2*×* C*H*_2_), 4.50–4.60 (m, 2H, 2*×* C*H*-N), 7.48–7.85 (m, 10H, 2Ph-*H* + 2CH=N), 8.24–8.32 (m, 6H, 2*×* pyr-*H*), 8.78 (m, 2H, 2*×* N*H*, exchangeable with D_2_O), 8.95 (m, 2H, 2*×* N*H*, exchangeable with D_2_O) and 10.18 (m, 2H, 2*×* N*H*, exchangeable with D_2_O). ^13^C-NMR: 20.32 (CH_3_), 27*.*45, 30.32, 38.64 (*6CH*_2_), 52.18 (2*CH*NH), 147.08 (*2CH*=N), 124.96, 125.05, 137.08, 137.10, 148.14, 148.18 (10pyr-*C*), 125.80, 128.05, 129.30, 139.48 (12Ar-C), 163.75, 169.77 (4*CO*NH), 172.15 (*2CO*-hydrazone). MS, *m/z* (%): 759 [M^+^, 8], 492 (100), 464 (15), 436 (25), 351 (9), 218 (78); Elemental analysis: calcd. for C_40_H_42_N_10_O_6_ (758.82): C, 63.31; H, 5.58; N, 18.46. found: C, 63.26; H, 5.51; N, 18.42.

*4,20-Di[oxo-2-methoxyphenylcarbohydrazonylmethyl)-3,8,16,21,27,28-hexaaza-2,9,15,22-tetraoxotri-cyclo[3,21,1,1^10,14^]octacosa-1(26),10,12,14,23,25-hexene* (**6h**): IR (KBr, cm^−1^): n 3338 (NH, amide), 1640 (C=N), 1662, 1552, 1320 (amide I, II and III). ^1^H-NMR (DMSO-*d*_6_): δ 1*.*34–1.38 (m, 4H, 2*×* C*H*_2_), 1.62–1.68 (m, 4H, 2*×* C*H*_2_), 3.18–3.25 (m, 4H, 2*×* C*H*_2_), 3.78 (s, 6H, 2 × OCH_3_), 4.45–4.55 (m, 2H, 2*×* C*H*-N), 7.36–7.76 (m, 10H, 2Ph-*H* + 2CH=N), 8.20–8.32 (m, 6H, 2*×* pyr-*H*), 8.88 (m, 2H, 2*×* N*H*, exchangeable with D_2_O), 9.10 (m, 2H, 2*×* N*H*, exchangeable with D_2_O) and 10.32 (m, 2H, 2*×* N*H*, exchangeable with D_2_O). ^13^C-NMR: 27*.*45, 30.68, 38.82 (*6CH*_2_), 52.48 (2*CH*NH), 55.14 (2C, 2OCH_3_), 147.30 (*2CH*=N), 124.86, 125.02, 136.95, 137.04, 148.06, 148.18 (10pyr-*C*), 112.75, 115.86, 120.86, 131.14, 132.05, 156.95 (12Ar-C), 163.68, 170.08 (4*CO*NH), 172.55 (*2CO*-hydrazone). MS, *m/z* (%):790 [M^+^, 15], 657 (12), 641 (45), 528 (22), 379 (95), 351 (35), 218 (100), 149 (18); Elemental analysis: calcd. for C_40_H_42_N_10_O_8_ (790.82): C, 60.75; H, 5.35; N, 17.71. found: C, 60.70; H, 5.30; N, 17.68.

*4,20-Di[oxo-4-methoxyphenylcarbohydrazonylmethyl)-3,8,16,21,27,28-hexaaza-2,9,15,22-tetraoxotri-cyclo[3,21,1,1^10,14^]octacosa-1(26),10,12,14,23,25-hexene* (**6i)**: IR (KBr, cm^−1^): n 3346 (NH, amide), 1642 (C=N), 1662, 1555, 1319 (amide I, II and III). ^1^H-NMR (DMSO-*d*_6_): δ 1*.*28–1.35 (m, 4H, 2*×* C*H*_2_), 1.60–1.67 (m, 4H, 2*×* C*H*_2_), 3.20–3.26 (m, 4H, 2*×* C*H*_2_), 3.68 (s, 6H, 2 × OCH_3_), 4.44–4.58 (m, 2H, 2*×* C*H*-N), 7.58–7.90 (m, 10H, 2Ph-*H* + 2CH=N), 8.22–8.30 (m, 6H, 2*×* pyr-*H*), 8.84 (m, 2H, 2*×* N*H*, exchangeable with D_2_O), 9.06 (m, 2H, 2*×* N*H*, exchangeable with D_2_O) and 10.24 (m, 2H, 2*×* N*H*, exchangeable with D_2_O). ^13^C-NMR: 27*.*52, 30.62, 38.74 (*6CH*_2_), 52.22 (2*CH*NH), 54.66 (2C, 2OCH_3_), 147.12 (*2CH*=N), 125.05, 125.10, 137.10, 137.14, 148.16, 148.24 (10pyr-*C*), 113.98, 125.64, 129.68, 162.62 (12Ar-C), 163.82, 169.76 (4*CO*NH), 172.25 (*2CO*-hydrazone). MS, *m/z* (%):790 [M^+^, 24], 657 (9), 641 (76), 528 (12), 379 (100), 351 (45), 218 (78), 149 (68); Elemental analysis: calcd. for C_40_H_42_N_10_O_8_ (790.82): C, 60.75; H, 5.35; N, 17.71. found: C, 60.69; H, 5.31; N, 17.67.

*4,20-Di[oxo-2,3,5-trimethoxyphenyl-carbohydrazonylmethyl)-3,8,16,21,27,28-hexaaza-2,9,15,22-tetraoxotricyclo[3,21,1,1^10,14^]octacosa-1(26),10,12,14,23,25-hexene* (**6j**): IR (KBr, cm^−1^): n 3336 (NH, amide), 1640 (C=N), 1662, 1556, 1322 (amide I, II and III). ^1^H-NMR (DMSO-*d*_6_): δ 1*.*32–1.36 (m, 4H, 2*×* C*H*_2_), 1.58–1.65 (m, 4H, 2*×* C*H*_2_), 3.18–3.28 (m, 4H, 2*×* C*H*_2_), 3.72 (s, 18H, 6 × OCH_3_), 4.34–4.42 (m, 2H, 2*×* C*H*-N), 7.25 (s, 4H, 2Ph-H), 7.88 (s, 2H, 2CH=N), 8.26–8.34 (m, 6H, 2*×* pyr-*H*), 8.92 (m, 2H, 2*×* N*H*, exchangeable with D_2_O), 9.14 (m, 2H, 2*×* N*H*, exchangeable with D_2_O) and 10.16 (m, 2H, 2*×* N*H*, exchangeable with D_2_O). ^13^C-NMR: 27*.*46, 30.68, 38.66 (*6CH*_2_), 52.18 (2*CH*NH), 55.16 (4C, 4OCH_3_), 58.78 (2C, 2OCH_3_), 147.24 (*2CH*=N), 124.98, 125.02, 137.15, 137.18, 148.10, 148.14 (10C, pyr-*C*), 105.12, 127.35, 140.64, 152.76 (12Ar-C), 163.86, 169.69 (4*CO*NH), 172.22 (*2CO*-hydrazone). MS, *m/z* (%): 910 [M^+^, 15], 777 (8), 701 (12), 673 (25), 692 (76), 436 (58), 303 (22), 237 (100), 218 (78); Elemental analysis: calcd. for C_44_H_50_N_10_O_12_ (910.92): C, 58.01; H, 5.53; N, 15.38. found: C, 57.95; H, 5.48; N, 15.33.

### 3.3. Antimicrobial Testing

The antimicrobial activity for the tested compounds was measured at 50 μg/mL, and was determined by the agar diffusion method as recommended by the National Committee for Clinical Laboratory Standards (NCCLS) [28,29,30]. The degree of inhibition is measured in mm in comparison with that of ampicillin and ketaconazole at the same concentration taken as standard values. 

In this method, two agar media, nutrient agar for bacteria and Czapek Dox agar for fungi were prepared and sterilized by autoclaving at 120 °C and 1.5 atm. for 20 min. The agar plates were poured left to cool down and after solidification they were inoculated with the bacterial and fungal strains by streaking. The tested compounds were loaded on sterile filter paper discs (5 mm diameter) at 50 µg/mL of DMSO and transferred aseptically into the inoculated agar plates along with ampicillin and ketaconazole discs at the same concentration for comparison. The plates were then incubated at 37 °C for 24 h for bacteria and at 30 °C for 48–72 h for fungi. After incubation, the diameters of the inhibition zones around the paper discs were measured in mm as an indication of the antimicrobial activities of the compounds.

## 4. Conclusions

A series of chiral macrocyclic imides and Schiff-bases **4**–**6** were synthesized using as starting material the macrocyclic bis-hydrazide derivative **3** [13], obtained from the corresponding ester **2** according to the published procedure [23,24]. The structure assignments of the new compounds are based on chemical and spectroscopic evidence. The newly synthesized compounds have been tested for their preliminary antimicrobial activity against Gram-positive bacteria, Gram-negative bacteria, and fungi. From the results it appears that some of the synthesized compounds have modest antimicrobial activities. The activities of all tested compounds against Gram positive bacteria (*B. subtilis* and *S. aureus*) are higher than that of the reference drug except for **6c**, **6g** (against *B. subtilis*) and **6d**, **6e**, **6h**, **6i** against *S. aureus.* The activities of compounds **3**, **4**, **5a**,**b**, **6b**, **6d**, and **6g**–**j** against the Gram negative bacterium *E. coli* were similar to that of the reference drug, while compounds **5b**, **6a**, **6c**, **6e** and **6f** are not active against the same organism. In addition, the compounds which were tested against fungal organisms (*C. albicans* and *A.niger*) had lower activities than the reference drug, or, as in the case of compounds **5b**, **6a**, **6b**, **6d**, **6e** and **6i**, were not active against the *C. albicans* strain.
